# Design, Synthesis and Characterization of Novel Co-Polymers Decorated with Peptides for the Selective Nanoparticle Transport across the Cerebral Endothelium

**DOI:** 10.3390/molecules23071655

**Published:** 2018-07-06

**Authors:** Andrea P. Falanga, Pietro Melone, Roberta Cagliani, Nicola Borbone, Stefano D’Errico, Gennaro Piccialli, Paolo A. Netti, Daniela Guarnieri

**Affiliations:** 1 Department of Pharmacy, University of Naples Federico II, 80131 Napoli, Italy; andreapatrizia.falanga@unina.it (A.P.F.); nicola.borbone@unina.it (N.B.); stefano.derrico@unina.it (S.D.); picciall@unina.it (G.P.); 2 Center for Advanced Biomaterials for Healthcare, Istituto Italiano di Tecnologia (IIT@CRIB), Largo Barsanti e Matteucci, 53, I-80125 Napoli, Italy; pietro.melone@unina.it (P.M.); paolo.netti@iit.it (P.A.N.); 3 Interdisciplinary Research Centre on Biomaterials, (CRIB), University of Naples Federico II, I-80125 Napoli, Italy; 4 Department of Chemical, Materials and Industrial Production Engineering, University of Naples Federico II, I-80125 Napoli, Italy; 5 Nanobiointeractions & Nanodiagnostics, Istituto Italiano di Tecnologia (IIT), Via Morego, 30, I-16163 Genova, Italy; roberta.cagliani@iit.it

**Keywords:** nanoparticles, peptides, blood-brain barrier, drug delivery, transport

## Abstract

The development of new strategies for enhancing drug delivery to the brain represents a major challenge in treating cerebral diseases. In this paper, we report on the synthesis and structural characterization of a biocompatible nanoparticle (NP) made up of poly(lactic-co-glycolic acid) (PLGA)-polyethylene glycol (PEG) co-polymer (namely PELGA) functionalized with the membranotropic peptide gH625 (gH) and the iron-mimicking peptide CRTIGPSVC (CRT) for transport across the blood-brain barrier (BBB). gH possesses a high translocation potency of the cell membrane. Conversely, CRT selectively recognizes the brain endothelium, which interacts with transferrin (Tf) and its receptor (TfR) through a non-canonical ligand-directed mechanism. We hypothesize that the delivery across the BBB of PELGA NPs should be efficiently enhanced by the NP functionalization with both gH and CRT. Synthesis of peptides and their conjugation to the PLGA as well as NP physical-chemical characterization are performed. Moreover, NP uptake, co-localization, adhesion under dynamic conditions, and permeation across in vitro BBB model are evaluated as a function of gH/CRT functionalization ratio. Results establish that the cooperative effect of CRT and gH may change the intra-cellular distribution of NPs and strengthen NP delivery across the BBB at the functionalization ratio 33% gH–66% CRT.

## 1. Introduction

The treatment of cerebral diseases such as brain tumors, HIV encephalopathy, epilepsy, cerebrovascular diseases, and neurodegenerative disorders is severely hindered due to the presence of the blood-brain barrier (BBB). The role of this barrier is to preserve brain homeostasis and to prevent toxic substances and invading organisms from reaching the brain. Therefore, an important feature of this barrier is the low and selective permeability to molecules, which excludes therapeutics from entering the brain parenchyma [1]. To date, the most common strategies to overcome this problem include drug administration directly into the CNS such as the temporary and local breakage of the endothelium, intracerebral injections, or the introduction of a slow-release implant into the brain [2]. Nanomedicine applied to the treatment of the CNS diseases provides a promising alternative strategy to conventional invasive and low effective brain treatments. In particular, the targeted-delivery of drugs to their action site by using nanocarriers such as NPs, dendrimers, nanogels, and nanoemulsions is able to combine the advantages of tissue targeting, drug loading, passing of BBB, and the reduction of side effects [3]. Usually, NPs enter the cell by using the endocytic pathway [4]. After endocytosis, the endosomes fuse with lysosomes where the acidification (pH < 5.5) allows either the degradation of the endosomes content or the delivery of endosomes cargo across the BBB [5]. In order to escape the endo-lysosomal pathway and to deliver the cargo across the BBB, the physical-chemical modulation of the NP surface plays a major role in mediating their interactions with the cell membrane including their intracellular fate. A key mechanism to improve the transport of NPs is by adding bioactive peptides such as the cell penetrating peptides (CPPs) to their surface. CPPs are short peptides of 5–40 amino acids provided with a net positive charge, helical moment, and amphipathic nature. These peptides enter the cells presumably by recognizing their cationic groups by negatively charged residues exposed on the cell membrane. Alternatively, membranotropic CPPs can efficiently cross the biological membranes by promoting the reorganization of plasma membrane lipid domains, which causes a temporary increase of membrane permeability [6,7,8]. Membranotropic CPPs take advantage of non-toxic components of neurotoxins to overcome the BBB reaching the CNS, which circumvents endosomal entrapment [9]. Among these, the gH peptide, which is derived from the glycoprotein of the Herpes simplex virus type 1, merges with model membranes and is able to traverse the membrane bilayer and to transport a cargo by escaping the degradation pathway of lysosomes [6,10,11]. In particular, in previous works, we demonstrated that the gH membranotropic peptide facilitates the delivery of polystyrene NPs across the murine BBB endothelium, which leads to a significantly higher cellular uptake by crossing through a non-conventional path [11] both in static [12] and dynamic [13] conditions. Moreover, in the case of metallic platinum ultrasmall NPs, gH partially increases their cytosolic delivery and anti-oxidant properties in Hela cells [14]. Furthermore, we showed that the peculiar mechanism of the entrance of NPs into the cells can easily be translated to soft matter nano-carriers such as oil/water nano-emulsions to enhance the cytosolic delivery of curcumin [15].

**Scheme 1:**
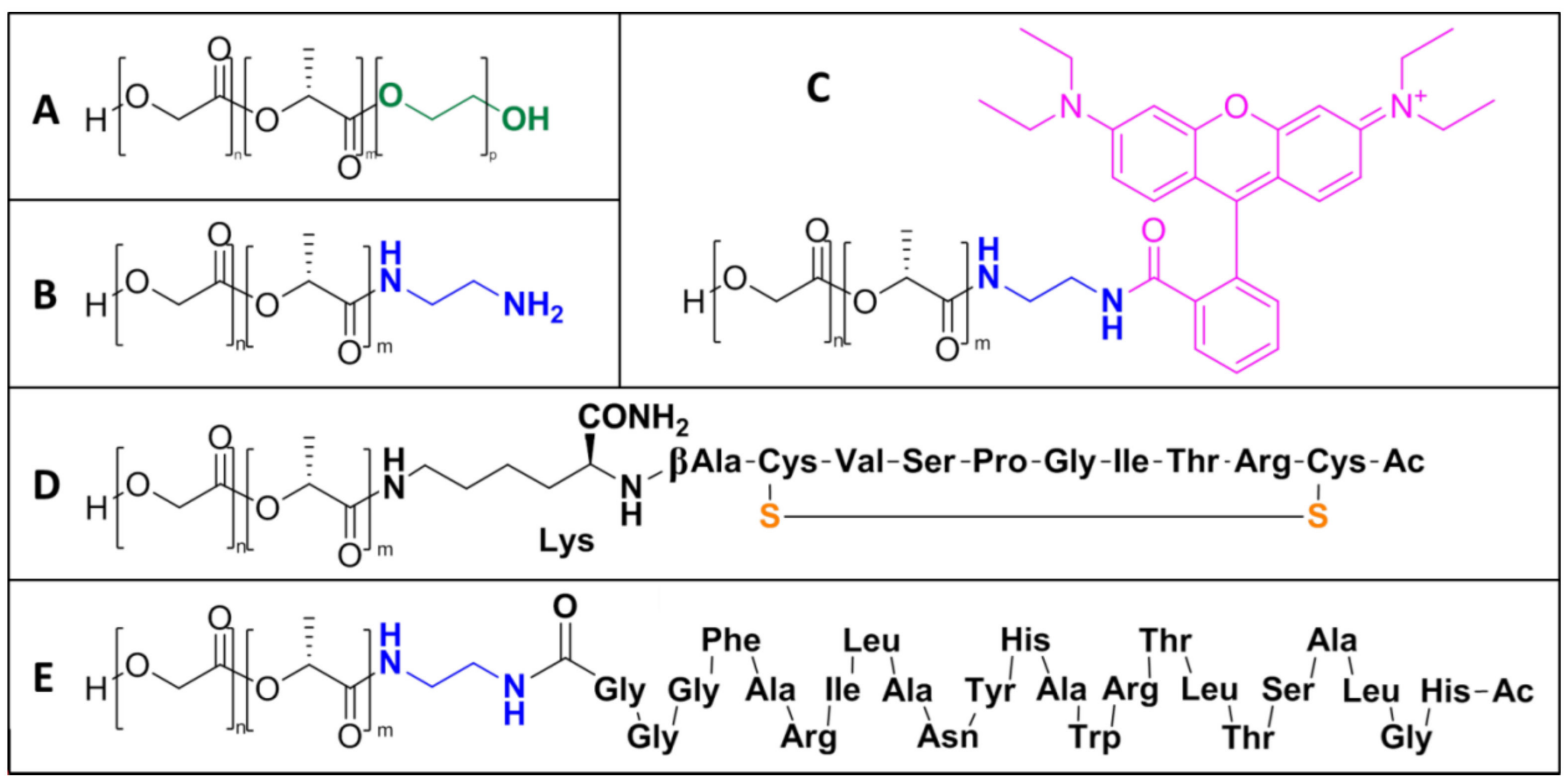
PLGA co-polymers and conjugates: (**A**) PLGA-PEG (PELGA) co-polymer, (**B**) PLGA-amine, (**C**) PLGA-rhodamine, (**D**) PLGA-CRT, and (**E**) PLGA-gH.

In this work, in order to translate the gH-based delivery platform to biodegradable systems made up of FDA-approved materials, we synthesized PELGA-NPs functionalized with gH, which has never been reported before in a BBB in vitro analysis. Due to their non-specific affinity to different cells, gH-mediated brain delivery systems could show a non-specific distribution in the human body after systemic administration. To overcome this drawback, the synthesis of nanocarriers functionalized with specific receptor-mediated transcytosis (RMT) ligands offers the possibility to target the cerebral endothelium by recognizing specific receptors overexpressed on the cells. The exploitation of RMT ligands such as Tf, insulin, and low density lipoprotein (LDL) has already been proposed for the transport of biologics into the brain, which allows NP delivery to the CNS [16,17,18,19]. The dual functionalization of PELGA NPs has been previously investigated [20,21,22]. Usually, specific ligands have high affinity for selective receptors, which promotes high cellular uptake of NPs through endocytosis mechanisms. However, often they are inefficient for enhancing transcytosis of NPs likely because of their endo-lysosomal compartmentalization. In fact, the main limitation of RMT is associated with low efficiency of BBB crossing and achievement into the CNS [23]. To date, only a few studies have clearly demonstrated the therapeutic effect of targeted nanocarriers in treating cerebral diseases [24,25]. Researchers have focused substantial efforts on identifying new BBB RMT targets. The most common method to identify them is through the screening of combinatorial peptide libraries. Staquicini et al. identified a new sequence, CRT (CRTIGPSVC), which is able to selectively target the brain endothelium and enhance brain crossing of viral particles [26]. CRT is a cyclic iron-mimicking peptide used as RMT-targeting ligand. CRT binds to apo-Tf causing it to adopt its iron-bound holo-Tf conformation, which recognizes TfR and gains access to the brain. TfR is highly expressed on immature erythroid cells, placental tissue, and rapidly dividing cells, which are both normal and malignant. Furthermore, it is expressed on hepatocytes and endothelial cells of the BBB [27,28]. Kang et al. demonstrated that the surface functionalization with the CRT peptide is able to specifically transport PELGA NPs across the BBB in vitro and in vivo [29]. Zhang et al. demonstrated that the conjugation of CRT on the surface of PLA-PEG NPs promotes cellular uptake and in vivo brain accumulation [30]. In this work, in order to avoid nanoparticle accumulation inside lysosomal compartments and overcome the gH lack of targeting specificity, the nanoparticles were functionalized with both gH and CRT peptides. Utilized NPs were prepared by mixing two polymeric species, i.e., the PELGA (Scheme 1A) and the functionalized PLGA-x where x is gH, CRT, rhodamine, or ethylenediamine (respectively E, D, C, B Scheme 1). We prepared and tested dual-functionalized NPs contained on the PLGA fraction including both the gH and CRT peptide tails. Three different gH/CRT ratios were synthesized: (i) 50%–50% (ii) 33%–66% (iii) 66%–33%. The hydrodynamic diameter and Z-potential, as well as the composition and functionalization of all the NPs prepared and tested are reported in Table 1 and Table 2, respectively. The effects of the functionalization on the targeting under dynamic conditions and the penetration of PELGA NPs through an in vitro BBB model based on the mouse brain endothelial bEnd.3 cells were evaluated.

## 2. Results

### 2.1. NP Building Blocks and NMR Analysis

Pure PLGA co-polymers and conjugates (Scheme 1) were synthesized and obtained in sufficient yield (from 80% to 85%). NP precursors were prepared through ester or amide linkage to the carboxylic group of PLGA, which optimized a previously reported synthesis [31,32]. In particular, Oxima Pure^®^ was used as an additive instead of 1-hydroxybenzotriazole (HOBt) both in peptide synthesis and in conjugation coupling reaction. It showed clear superiority to HOBt (which have been reported to exhibit explosive properties) [33] in terms of suppression of racemization, coupling efficiency, and safety [34]. With regard to PLGA-rhodamine (Scheme 1C) and PLGA-gH (Scheme 1E), an ethylenediamine linker between the two carboxylic groups of conjugates was previously added (PLGA-amine–Scheme 1B).

**Figure 1:**
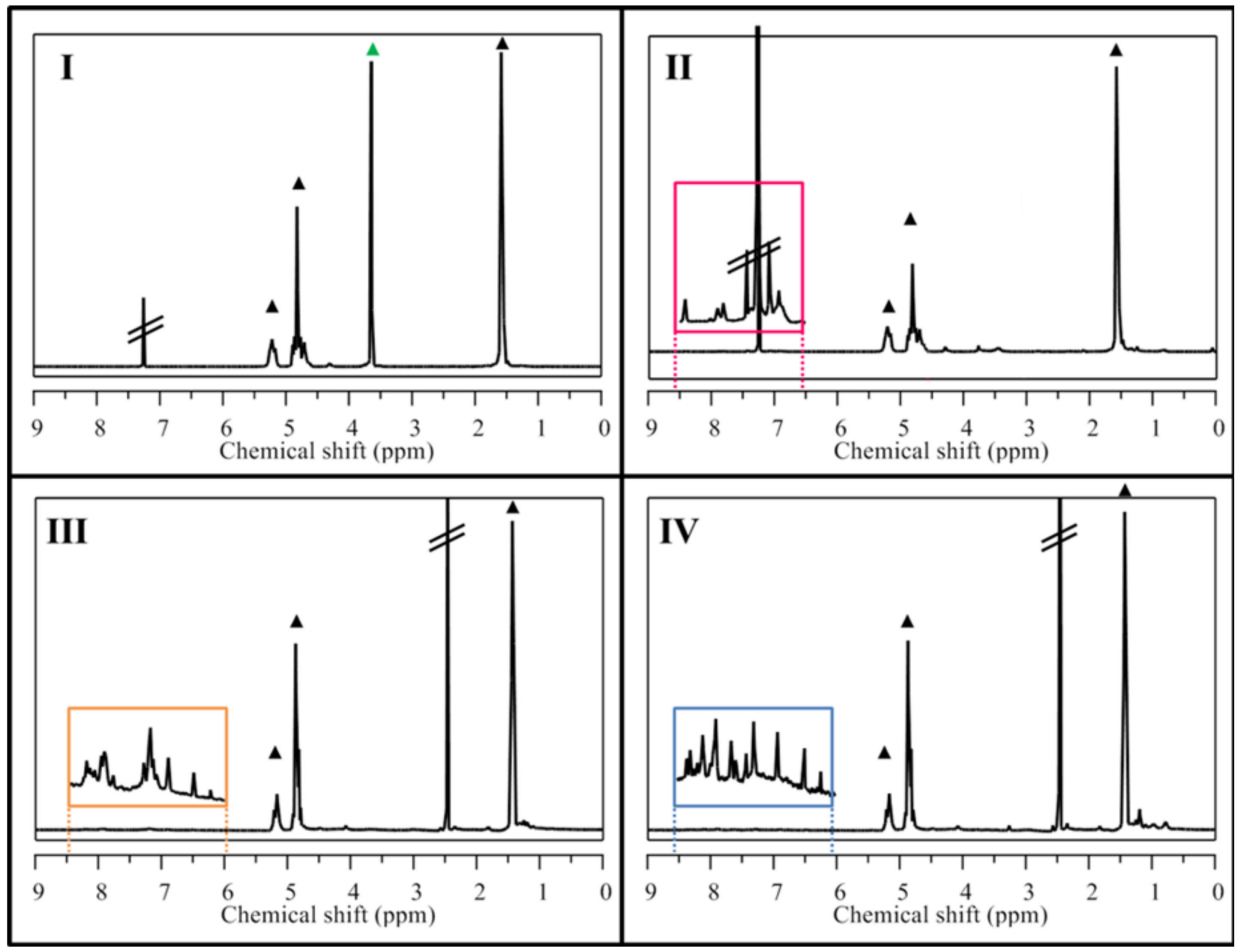
^1^H-NMR spectra of synthesized PLGA conjugates showed in Scheme 1. In every spectrum, peaks related to PLGA were marked with black triangles (▲). (**I**) PELGA co-polymer with PEG signal 3.64 ppm (▲), (**II**) PLGA-rhodamine with rhodamine aromatic signals highlighted in magenta expansion square, (**III**) PLGA-CRT and (**IV**) PLGA-gH with peptide amide backbone signals highlighted in orange and blue expansion squares, respectively.

The chemical characterization of synthesized NP precursors was carried out using two techniques. First, the peptide synthesis step and the CRT cyclization reaction were followed by LC-MS in order to confirm peptide sequences and disulfide bond formation (Figure S1). Then ^1^H-NMR spectroscopy was used after dialysis purification of the co-polymers in order to assess the products purity. The presence of proton signals belonging to the CRT and gH peptides (see the NH amide protons in the orange and blue squared insets, respectively, in Figure 1) or to the aromatic protons of rhodamine (purple and red squared inset in Figure 1) confirmed the success of the coupling step for all the PLGA conjugates.

### 2.2. NP Characterization

The prepared NPs were characterized in size and surface charge by DLS and Z-potential measurements in aqueous medium. The hydrodynamic diameter (H_D_) of NPs after filtration was found to be below 100 nm and ranged from 70 nm and 95 nm for all the particle formulations. Table 1 reports the H_D_ values of the different NPs. Moreover, all the suspensions showed polydispersity values of ca. 0.15, which indicates a narrow distribution of the particle size. Z-potential measurements indicated a negative surface charge around −20 mV for all NP types with slight differences among the formulations due to the presence of the peptides. A more positive charge was shown for gH NPs and this is probably due to the positive charge of the amino-acid chain. The stability of NP suspensions was monitored over time.

### 2.3. NP Intracellular Distribution

**Figure 2:**
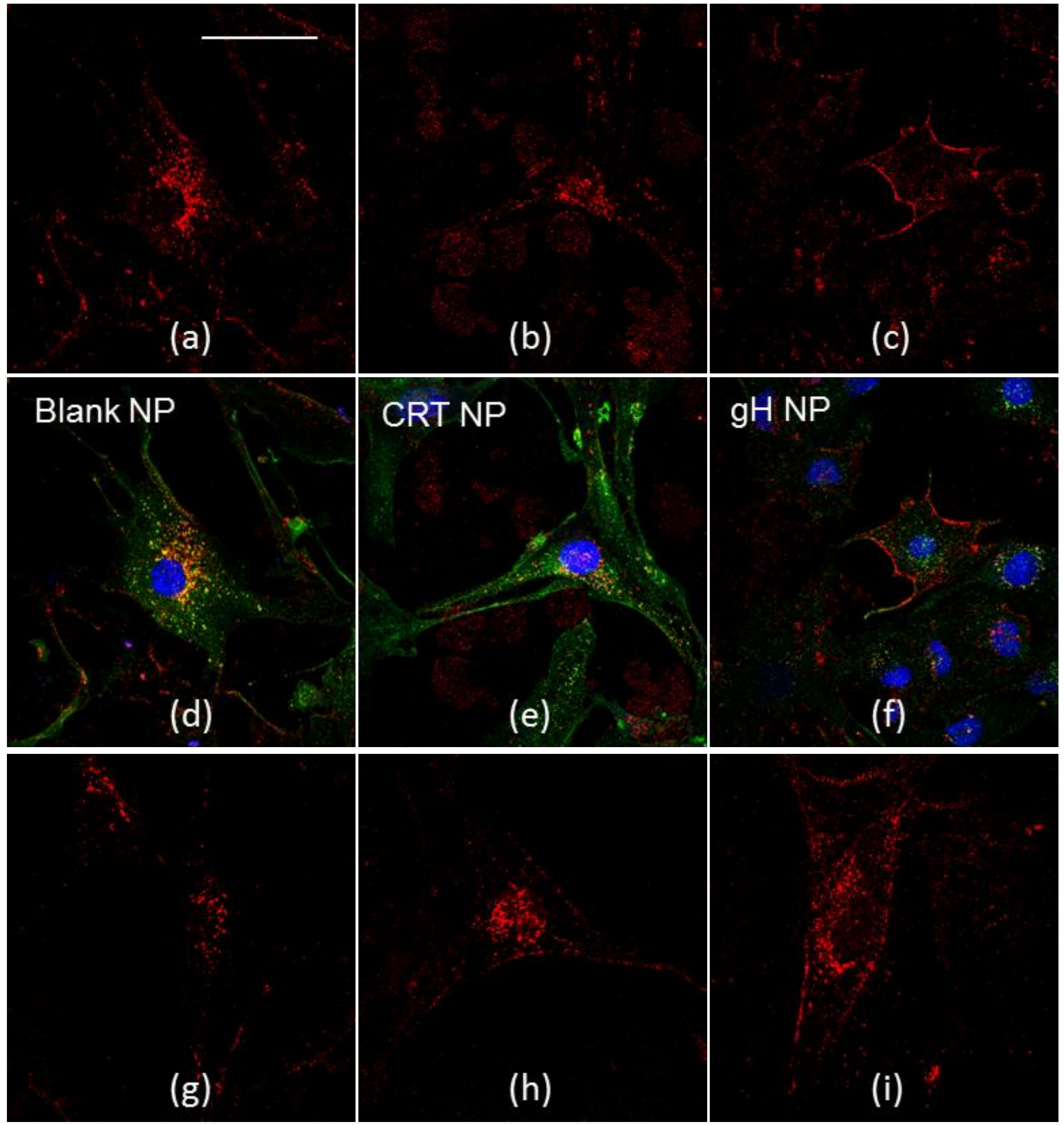

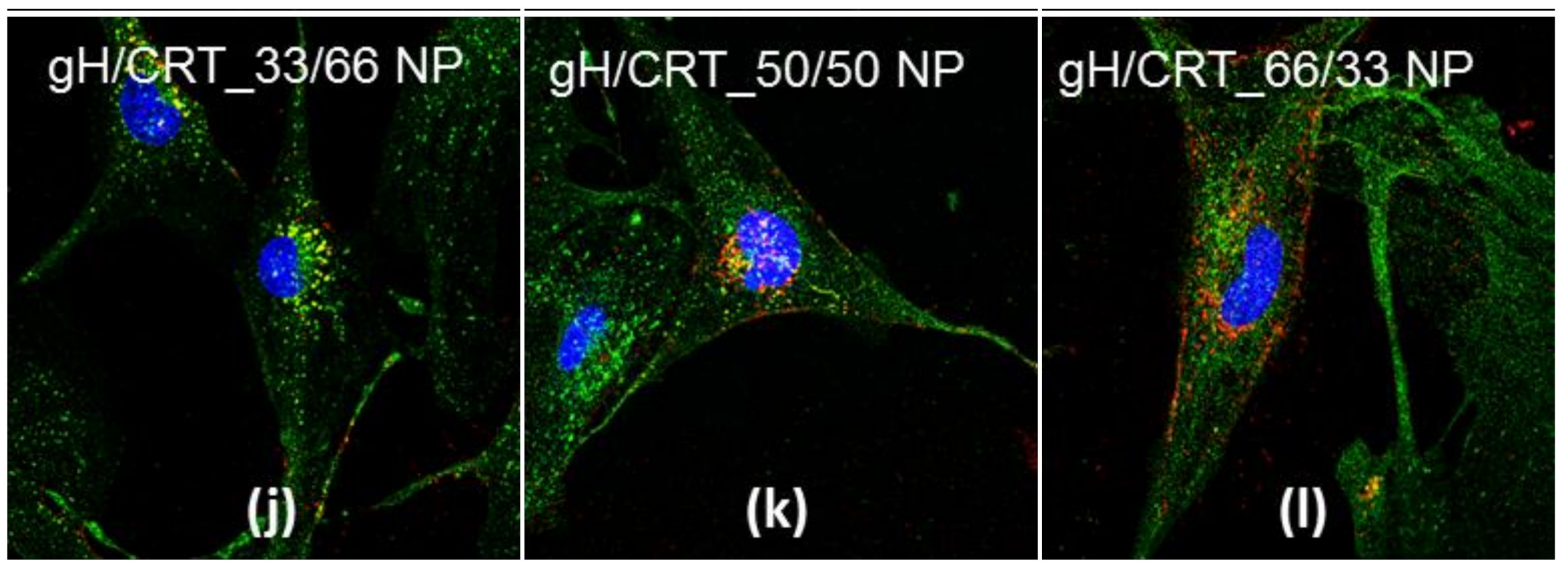
Co-localization of blank NPs (**a**,**d**), CRT NPs (**b**,**e**), gH NPs (**c**,**f**), gH/CRT_33/66 NPs (**g**,**j**), gH/CRT_50/50 NPs (**h**,**k**), gH/CRT_66/33 NPs (**i**,**l**) with lysosomes after 24 h of incubation in bEnd.3 cells. Red: NPs, green: LAMP-2, blue: nuclei. Bar 50 µm.

Indirect immunofluorescence against endocytic markers may give information about the endocytic mechanisms underlying nanoparticle cellular uptake. In order to investigate the intracellular distribution and fate of NPs, co-localization experiments with lysosomes were performed. After 24 hours of incubation, which is shown in Figure 2, blank, CRT, gH, gH/CRT_66/33, gH/CRT_50/50, and gH/CRT_33/66 NPs were internalized by the cells.

NPs showed different intracellular localization. Both blank and CRT NPs enter bEnd.3 cells and partially co-localize with LAMP-2 glycoprotein, which is a marker of lysosomal compartments. Conversely, gH NPs showed a main cytoplasmic distribution and no significant co-localization with lysosomes was noticed, according to previous studies [11,12]. Localization of dual-functionalized NPs changed as a function of the formulation. More precisely, lysosomal distribution increased with the amount of CRT in the formulation. Likewise, cytoplasmic distribution increased by increasing the gH amount. In this way, it became more similar to the mono-functionalized NPs.

### 2.4. NP Transport across the BBB Endothelium

**Figure 3:**
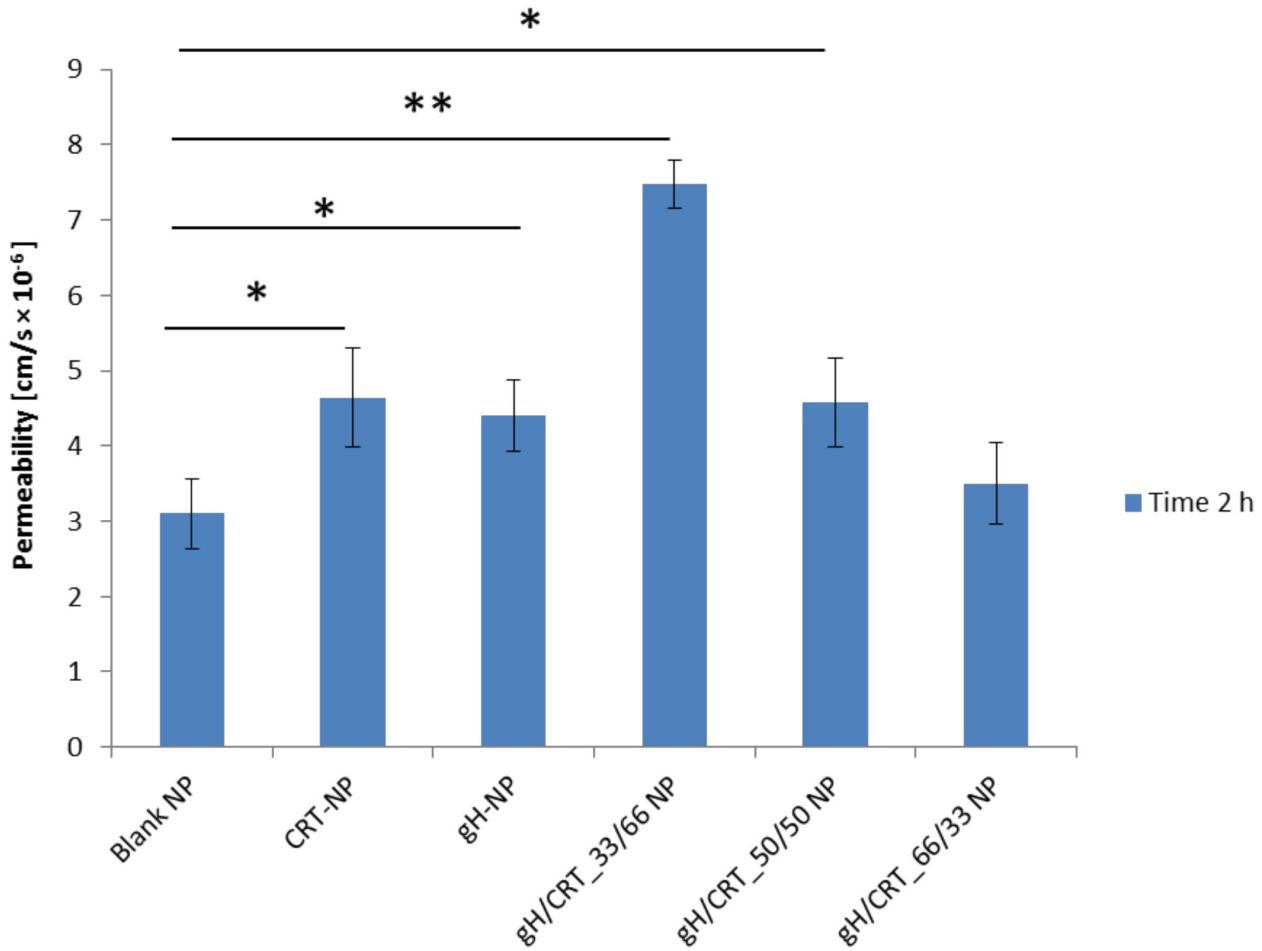
Permeability of bEnd.3 cell monolayer to blank, CRT, gH, gH/CRT_33/66, gH/CRT_50/50, and gH/CRT_66/33 functionalized NPs. * *p* = < 0.05, ** *p* = < 0.0005.

bEnd.3 cells formed a confluent monolayer that was able to mimic the properties of the BBB endothelium when cultured on porous inserts [35,36]. The development of tight junctions was followed by using TEER measurements. After 7 days of cell seeding, TEER was found to be 21.67 ± 4.94 Ω cm^2^ (*n* = 6), which reached a value comparable to reported studies [37]. Therefore, in order to study if the functionalization could affect the ability to cross the in vitro BBB, transport experiments through the bEnd.3 confluent monolayer were performed. Data reported in Figure 3 demonstrated that CRT NPs and gH/CRT_33/66 NPs crossed the endothelial layer more efficiently when compared to blank NPs.

In particular, among mono-functionalized NPs, CRT NPs showed the highest ability in NP transport across the in vitro BBB endothelium with a permeability of 4.64 × 10^−6^ cm/s. No significant increase in permeability of the barrier was found for gH functionalization (4.40 × 10^−6^ cm/s) compared to the blank NPs (3.17 × 10^−6^ cm/s). On the other hand, for the dual-functionalized NPs, the permeability increment grew by decreasing the CRT amount in the gH/CRT ratio. More precisely, permeability of the BBB endothelium was 7.48 × 10^−6^ cm/s for gH/CRT_33/66, 4.57 × 10^−6^ cm/s for gH/CRT_50/50 NPs, and 3.50 × 10^−6^ cm/s for gH/CRT_66/33. Therefore, gH/CRT_33/66 NPs showed the highest of trans-endothelial crossing ability. Permeability results suggested a cooperative effect on BBB crossing of gH and CRT peptides when exposed on NPs in percentages of 33% to 66%, respectively.

### 2.5. NP Adhesion to the BBB Endothelium in Flow Conditions

In vivo, the brain endothelium is exposed to the bloodstream. Therefore, in physiological conditions, NPs should recognize and bind the endothelial wall under dynamic forces before crossing the BBB. To address this issue, we investigated how the surface functionalization could affect the targeting ability of NPs to the cerebral endothelium by using a parallel plate flow chamber Glycotech system, which is a commercial device mimicking hydrodynamic conditions in the microcirculation. Among dual-functionalized NPs, gH/CRT_33/66 formulation was chosen for the dynamic adhesion assay due to the highest ability to transport the NPs across the in vitro BBB. The volumetric flow rate *Q* (10 µL/min) was fixed to be equal for all the experiments. Based on these data, the mean velocity is shown below.

(1) $$U=\frac{Q}{wh},$$

within the chamber, it was ≅0.066 mm/s. The shear rate is shown below.

(2) $$S=\frac{6Q}{h^{2}w},$$

this was 1.55 s^−1^ and the shear stress at the wall is shown below.

(3) $$\tau_{w\;}=µ\;S,$$

this was 1.86 × 10^−3^ Pa.

**Figure 4:**
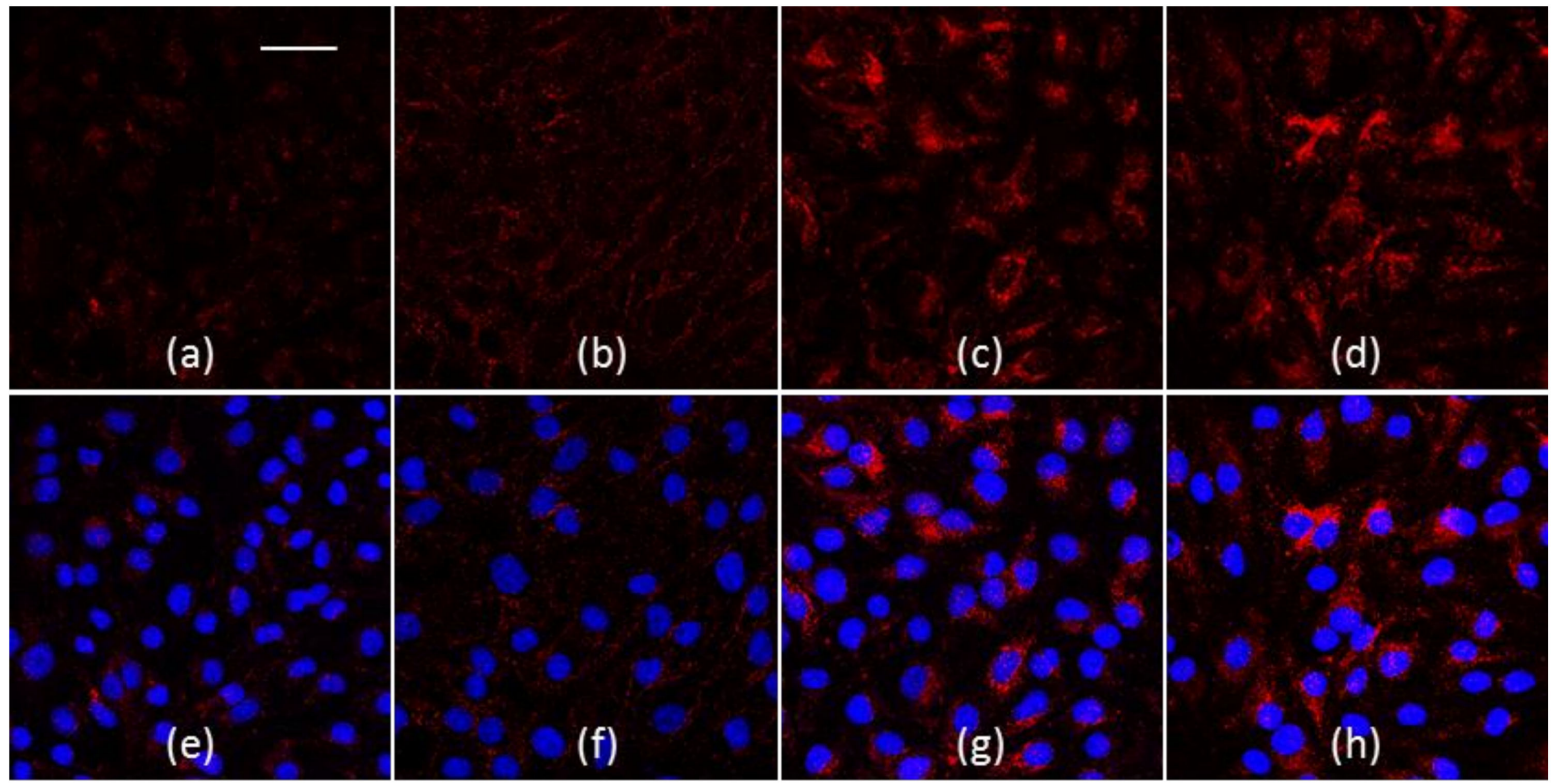

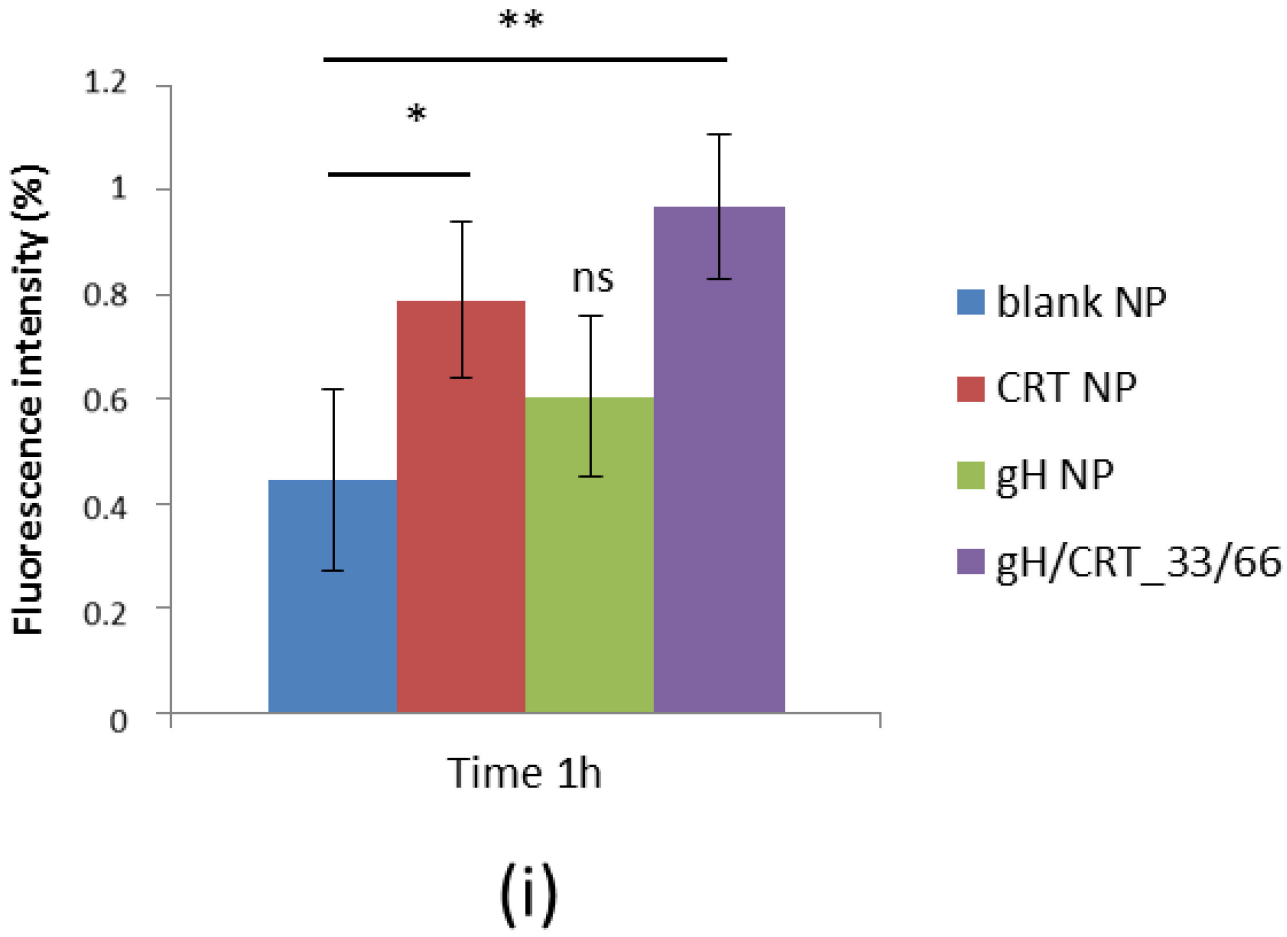
(**a**–**h**) Confocal images of NPs in bEnd.3 after a 1-hour experiment in flow conditions. (**a**,**e**) blank NPs, (**b**,**f**) gH NPs, (**c**,**g**) CRT NPs, (**d**,**h**) gH/CRT_33/66. Red: NPs, blue: nuclei. Bar 50 µm. (**i**) Percentage of fluorescence intensity of NPs adherent to bEnd.3 layer after 1 h under flow condition (flow rate 10 µL/min). * *p* < 0.05, ** *p* < 0.0005; ns = not significant.

The mean velocity used for the experiments was comparable with the blood velocity in the human capillary vessels that is calculated between 0.01 mm/s and 0.1 mm/s [38]. The shear rate and the shear stress at the wall were sufficiently small to allow adhesion of nanoparticles to endothelial cells [39]. The Reynolds number is clearly associated with a laminar flow and was 0.035. In Figure 4a–h, some representative confocal images of BBB endothelia after 1 hour of treatment with blank (Figure 4a,e), gH (Figure 4b,f), CRT (Figure 4c,g), and gH/CRT_33/66 (Figure 4d,h) NPs were reported. The images showed a higher red fluorescence related to NPs for cells treated with CRT and gH/CRT NPs than blank and gH NPs. To quantify NP adhesion, 10 confocal images of different areas of the endothelial layers treated with each NP type were acquired and analyzed by ImageJ software. Results of fluorescence image analysis demonstrated that, among mono-functionalized NPs, CRT NPs possessed the higher adhesion ability to the bEnd.3 monolayer compared to blank NPs. As shown in Figure 4i, the calculated fluorescence percentage of CRT NPs was 0.79% against 0.44% of blank NPs. Conversely, no significant increase in adhesion for gH NPs with a fluorescence percentage of 0.60% was found in comparison with blank NPs. For dual-functionalized gH/CRT_33/66 NPs, the percentage of adhesion was 0.97%. Therefore, adhesion assay results indicated that the presence of the gH peptide did not enhance significantly the targeting of the cerebral endothelium under dynamic conditions.

## 3. Discussion

The nanoparticle ability to recognize and cross the brain endothelium is an important requirement for successful brain therapies. The design of targeted-delivery carriers has garnered significant attention in recent years because of the potential to achieve highly localized delivery to brain endothelium [17,23]. The physical-chemical modulation of the surface of nanocarriers offers the possibility to restrict the biological effect of the therapy to a specific type of cells by avoiding the potentially toxic contact with cells not of interest. The challenge to win is the achievement of CNS, which reaches the threshold of a sufficient drug concentration in order for it to be effective, which avoids side effects to healthy tissues. To overcome these limitations, biodegradable PELGA nanoparticles, which provide good biocompatibility, high reproducibility, great pay-load properties, and easiness in functionalization were used as nanocarriers. In particular, we recently reported on the versatility of PELGA nanoparticles as efficient drug delivery systems [32,33,34,35,36,37,38,39]. In this work, PELGA NPs were synthetized by using the nanoprecipitation method and combining co-polymers and peptide-functionalized polymers as building blocks. This method allowed us to bypass the functionalization step, which reduces the time required for NP production. The data presented in this paper characterized a new delivery platform to promote brain penetration. Recent studies based on this perspective focused increasing attention on developing several strategies in order to deliver drugs across the BBB by combining targeting and crossing strategies. Zheng et al. designed double functionalized liposomes with Transferrin (Tf) and the CPP TAT to promote their translocation across the BBB [40,41]. Data of liposome biodistribution ex vivo showed that Tf/TAT liposomes accumulated the most in the brain when compared to controls. However, a non-specific accumulation in other organs such as lung and liver after 12 hours was also detected. Despite several studies proving the effectiveness of CPPs such as TAT in promoting the NP transport across the BBB [42], the TAT peptide demonstrated the involvement of the endocytosis pathway in the internalization mechanism [43,44,45], which allowed for the storage of the cargo inside endosomes and lysosomes. Therefore, only a small quantity of drugs are able to cross the cell and reach the brain. In this context, in order to overcome the limitation of TAT in the involvement of the endocytic pathway, we chose gH as CPP, which was widely characterized in previous works [11,12,13,14,15]. gH facilitates NP delivering across the BBB, which leads to a significant higher cellular uptake and crossing. It escapes the endo-lysosomal entrapment. Therefore, gH is able to overcome the limitations of TAT on the endocytic mechanism of internalization. As described above, in our previous validation studies, we showed that gH enters cells and is able to transport cargo across cells. For a targeted NP delivery, among the various methods used by researchers to recognize the cerebral endothelium, Tf/TfR is one of the most common strategies for active transport [46,47,48]. In order to target the cerebral endothelium, we chose the iron-mimicking peptide CRT, which is able to target the Tf/TfR complex. The contribution of both gH and CRT peptides in the trans-endothelial ability to cross the in vitro BBB in dual-functionalized NP formulations was tested and compared to blank and mono-functionalized nanoparticles. To this aim, we first performed cellular uptake and lysosomal co-localization experiments of gH/CRT co-modified NPs in three different ratios, which are 50/50, 33/66, and 66/33. These formulations were compared with CRT or gH mono-functionalized NPs and non-functionalized blank NPs. Confocal images showed that functionalized-NPs had different intra-cellular distributions. In particular, the presence of the gH peptide played a key role in controlling the NP intracellular fate. In fact, it reduced the lysosomal storage of NPs. The specific action of the gH peptide in decreasing NP accumulation in lysosomes was also confirmed by transport experiments across the in vitro BBB. Moreover, the presence of CRT promoted NP transport across the BBB, but the co-presence of gH was able to further enhance the crossing. This effect was shown when gH/CRT peptides were exposed in percentages of 33% or 66% on the NPs. Therefore, the NP transport results confirmed our initial hypothesis. The cooperative effect was not evident in the case of 50%/50% and 66%/33% NP formulations. Further investigations such as the analysis of conformation and exposure of peptides on NP surface are needed to elucidate the higher performance of the gH/CRT_33/66 NP formulation compared to others. gH is a 19 residues peptide with helical moment while CRT is a cyclic peptide of nine amino-acids. Likely, the lower concentration of gH in gH/CRT_33/66 NP formulation than gH/CRT_50/50 and gH/CRT_66/33 NPs allows for the reduction of the steric hindrance, which promotes the correct exposure of both peptides on the NP surface during the preparation. Therefore, the peptides had more conformational freedom and a higher ability to perform their proper function. We also investigated if the contribution of gH in increasing NP transport was due to an enhancement in the NP association to the endothelial cell layer by using an NP adhesion assay under dynamic conditions. Data from these assays indicated that the percentage of gH NPs was quite similar to blank NPs and the percentage of gH/CRT_33/66 was similar to CRT NPs. These findings clearly indicated that the presence of gH on the PELGA NP surface did not enhance the recognition of the endothelial cells. Therefore, it may be hypothesized that the enhancement in NP crossing cannot be ascribed to the targeting enhancement, but can be ascribed to the mechanism of the gH peptide which reduced lysosomal escaping and promoted NP transport across the in vitro BBB. However, in a prior study, we demonstrated that the decoration of polystyrene NPs with the gH625 peptide enhanced the adhesion of those particles to the endothelial layer and the BBB crossing in flow conditions [13]. This suggests an effect on the nature of the particle in affecting the functionality of cell-penetrating peptides. Taken altogether, our results demonstrated that the dual functionalization strategy based on the use of a cell membrane penetrating and a targeting peptide, which are known as gH and CRT, enhanced the recognition under static and dynamic conditions and the transport across the BBB endothelium of PELGA NPs.

## 4. Materials and Methods

### 4.1. Reagents and Materials

Equimolar uncapped poly(d,l-lactide-co-glycolide) (PLGA) (Resomer RG502H, average Mw 12 kDa) was purchased from Boehringer Ingelheim (Ingelheim, Germany). Polyethylene glycol (PEG, Mw 1500 Da), Ethylenediamine (en), *N*,*N*-diisopropylethylamine (DIPEA), *O*-benzotriazole-*N*,*N*,*N*′,*N*′-tetramethyluronium-hexafluoro-phosphate (HBTU), Ethyl 2-cyano-2-(hydroxyimino) acetate (Oxyma Pure Novabiochem®), anhydrous *N*,*N*-dimethyl-formamide (DMF), Acetonitrile (ACN), *N*,*N*′-Diisopropylcarbodiimide (DIC), *N*,*N*′-Dicycloexylcarbodiimide (DCC), Rhodamine B, 4-(dimethylamino) pyridine (DMAP), Dichloromethane (DCM), Dimethylsulfoxide (DMSO), Sodium carbonate (Na_2_CO_3_), Piperidine, Trifluoroacetic acid (TFA), Formic acid, HPLC-grade water, and all buffer solutions were purchased from Sigma-Aldrich S.r.l.(Milan, Italy). All Fmoc-aminoacids were purchased from IRIS Biotech GmbH (Marktredwitz, Germany). Dialysis bag MWCO 6000–8000 Da were purchased from Spectrum Europe B.V. (Breda, Netherlands). Distilled and deionized water used Millipore Milli-RO 10 Plus from Merck S.p.a. (Milan, Italy) at 18 MΩ resistance. Amicon^®^ Ultra-4 centrifugal filters were purchased from Merck S.p.a. (Milan, Italy).

### 4.2. Peptide Synthesis and Characterization

CRT (Ac-CRTIGPSVC-βAK-CONH_2_) and gH (Ac-HGLASTLTRWAHYNALIRAFGGG-COOH) peptides were synthesized using the standard solid-phase-9-fluorenyl methoxy carbonyl (Fmoc) procedure and were obtained with good overall yields (35%–40%). The β-Alanine-Lys (as regards CRT peptide) and the Gly_3_ (for gH625 peptide) residues acted as spacer units between polymer and peptide active sequences in order to retain the native peptide conformation when conjugated to the particle surface.

The syntheses were performed by using the Biotage® Syro Wave™ peptide synthesizer (Biotage, Uppsala, Netherland). Rink Amide and Wang resin was used as a solid-phase support for CRT and gH peptide, respectively. Previously, the C-terminal glycine residue (10 eq) of the gH peptide was manually attached to an equivalent amount of Wang resin using Oxima Pure^®^ (10 eq), DIC (10 eq), and DMAP (0.4 eq). The Fmoc protecting group was removed using piperidine 40% (*v*/*v*) solution in DMF. Peptides were cleaved from resin using the TFA/TIS/H_2_O (95/2.5/2.5) solution and precipitated in ice-cold diethyl ether. Purified CRT peptide was obtained by using RP-HPLC starting from 5/95 (*v*/*v*) acetonitrile (ACN)/water solution containing 0.1% *v*/*v* TFA. For the purification of the gH peptide, a 20/80 ACN/water solution with 0.1% *v*/*v* TFA was used. Peptides were lyophilized and then characterized using ESI-LC-MS. These analyses were carried out by injecting aqueous solutions of peptides into Agilent ZORBAX EclipsePlus C18 RRHD (1.8 µm, 2.1 × 50 mm) (Agilent, Santa Clara, CA, USA) eluted with solvent solution of H_2_O and ACN containing 0.1% *v*/*v* of formic acid. A linear gradient of 20%–80% of ACN over 8 min at a flow of 150 µL/min was used.

MS for the CRT peptide was calculated for [M + H]^+^ = 1176.43 *m*/*z*; found (ESI) [M + H]^+^ = 1175.78 *m*/*z*, [M + 2H]^2+^ = 588.43 *m*/*z*, [M + 3H]^3+^ = 392.64 *m*/*z*. MS for the gH peptide was calculated for [M + H]^+^ = 2512.79 *m*/*z*; found (ESI) [M + 2H]^2+^ = 1256.68 *m*/*z*, [M + 3H]^3+^ = 838.12 *m*/*z*, [M + 4H]^4+^ = 628.84 *m*/*z* (Supplementary Materials S1).

### 4.3. Peptide Cyclization

The purified CRT peptide was cyclized by the air-oxidation method to allow the formation of the intra-chain disulfide bond between the two cysteine residues. Briefly, the cysteinyl peptide (0.1 mg/mL) was dissolved in 0.1 M sodium carbonate (pH 8) and left to stand open to the atmosphere under vigorous magnetic stirring until the reaction was complete (48 h). The reaction progress was monitored by the LC-MS analysis. MS for CRT-cyclized peptide was calculated for [M + H]^+^ = 1174.43 *m*/*z*. It was found that (ESI) [M + H]^+^ = 1173.77 *m*/*z*, [M + 2H]^2+^ = 587.43 *m*/*z*, and [M + 3H]^3+^ = 391.97 *m*/*z*. Lastly, the CRT-peptide was purified and lyophilized as described above. The product was obtained with 86% yield.

### 4.4. Synthesis of Co-Polymers and Conjugates

#### 4.4.1. PELGA

PLGA-PEG co-polymer (namely PELGA) was synthesized via a coupling reaction between PLGA and PEG [1]. The carboxyl group of PLGA reacted with the terminal hydroxyl group of PEG. 1 eq of PLGA, 4 eq of PEG, 0.4 eq DMAP, and 2 eq of DCC were dissolved in 10 mL of anhydrous DCM. After the reaction (2 days, RT, inert atmosphere), the residual DCC was changed into dicyclohexylcarbodiurea (DCU) by adding 10 μL of bi-distilled water. Then DCM was evaporated and the mixture was dissolved in 10 mL of DMSO and then filtrated and dialyzed (MWCO 6–8 kDa) for 1 day against ACN and for 2 days against water. The pure product was recovered after lyophilization and its identity was confirmed by using ^1^H-NMR spectroscopy (600 MHz, CDCl_3_): δ ppm 5.30–5.13 (PLGA, CH), 4.92–4.60 (PLGA, CH_2_), 3.64 (PEG, CH_2_), 1.59 (PLGA, CH_3_).

#### 4.4.2. PLGA-Amine

PLGA was functionalized with ethylenediamine (en), which acts as a bridge between PLGA and rhodamine (or peptide C-terminal) carboxylic groups. In addition, 30 mg of PLGA 502 h, 10.3 mg of DCC, 8.7 µL of DIPEA, 10.0 µL of ethylenediamine were dissolved in 2.5 mL of anhydrous DCM. After the reaction (1 day, RT, inert atmosphere), the residual DCC was changed into dicyclohexylcarbodiurea (DCU) by adding 10 µL of bi-distilled water. The solution was filtered, precipitated dropwise into cold methanol, centrifuged, and then placed under vacuum overnight. The identity of the pure product was confirmed by using ^1^H-NMR spectroscopy (600 MHz, DMSO-*d*_6_): δ ppm 8.13 (en, amide), 5.26–5.10 (PLGA, CH), 4.95–4.75 (PLGA, CH_2_), 3.14 (2 × CH_2_ overlapped, en), 1.44 (PLGA, CH_3_).

#### 4.4.3. PLGA-Rhodamine

The PLGA-Rhodamine conjugation was performed by a standard coupling procedure. PLGA-amine (1 eq), HBTU (5 eq), DIPEA (10 eq), and rhodamine-B (5 eq) reacted in anhydrous DMSO for 24 h at RT and then purified by dialysis bags (MWCO 6–8 kDa) against water for 3 days. ^1^H-NMR (600 MHz, CDCl_3_): δ ppm 8.42, 7.85, 7.05, and 6.92 (rhodamine aromatic proton), 5.30–5.13 (PLGA, CH); 4.92–4.60 (PLGA, CH_2_), 4.30 (rhodamine CH_2_), 3.78 (rhodamine CH_3_), 3.42 (en, CH_2_), 1.59 (PLGA, CH_3_).

#### 4.4.4. PLGA-Peptide Conjugates

PLGA-Peptide conjugates were synthesized using a standard HBTU coupling procedure. For the CRT peptide, PLGA (1 eq), HBTU (5 eq), Oxima Pure^®^ (5 eq), DIPEA (10 eq), and Peptide (1.5 eq) were dissolved in anhydrous DMSO for 48 h at RT. For the gH peptide, the PLGA-amine 2 eq of peptide was used. Adducts were purified from unreacted reagents using dialysis bags (MWCO 6–8 kDa) against pure water and lyophilized. The reaction products were confirmed by using ^1^H-NMR spectroscopy. PLGA-CRT: ^1^H-NMR (600 MHz, DMSO-*d*_6_): δ ppm 8.50–6.00 (peptide amide backbone), 5.28–5.14 (PLGA, CH), 4.96–4.84 (PLGA, CH_2_), and 1.47 (PLGA, CH_3_). PLGA-gH: ^1^H-NMR (600 MHz, DMSO-*d*_6_): δ ppm 8.50–6.00 (peptide amide backbone), 5.28–5.14 (PLGA, CH), 4.96–4.84 (PLGA, CH_2_), 3.30 (en, CH_2_), and 1.47 (PLGA, CH_3_).

### 4.5. Nuclear Magnetic Resonance (NMR) Analysis

All Nuclear Magnetic Resonance (NMR) spectra were recorded at 28 °C using an Agilent 600 MHz (14 T) spectrometer equipped with a DD2 console and a OneNMR HX probe. The samples (1 mg) were dissolved in 600 μL of 99.9% deuterated solvent (Sigma–Aldrich). ^1^H 1D spectra were recorded using 256 scans to obtain a good signal-to-noise ratio for peptides, en and rhodamine. A saturation PRESAT pulse sequence was used to reduce residual peaks of water at 3.33 ppm. Spectra were transformed and analyzed using VNMRJ 4 software and the chemical shift scale was referenced to the solvent residual peak signal.

### 4.6. NP Preparation

Rhodaminated PELGA-Peptide nanoparticles were prepared by using the nanoprecipitation method [32].

In summary, the proper amounts of co-polymers and conjugates (Table 2) were dissolved in acetone and mixed. The final volume was adjusted to 1.3 mL. Afterwards, the solution was added dropwise (6 mL/h) with a syringe pump into 12.5 mL of distilled water under magnetic stirring (400 rpm). The solution was kept under magnetic stirring until complete evaporation of the organic solvent (3–5 h) was obtained. The obtained NP suspension was sterilized with a 0.22 μm membrane filter. Lastly, the volume of the solution was reduced to 1 mL using Amicon^®^ Ultra-4 centrifugal filter. The final NP concentration (mg/mL of polymeric species) was estimated by fluorescence intensity measurements of rhodamine-PLGA using a Victor 2 spectrofluorometer (Perkin Elmer Wallac, Turku, Finland) that were compared to fluorescence intensity of the initial NP concentration before filtration. Three different batches of NP formulation were prepared for all the experiments.

### 4.7. Cell Culture

Immortalized mouse cerebral endothelial bEnd.3 cells (American Type Culture Collection, Manassas, VA, USA) were grown at 37 °C and 5% CO_2_ in DMEM with 4.5 g/L w/o phenol red (Gibco Thermo Fisher, USA), supplemented with 10% FBS, 3.7 g/L sodium bicarbonate and 4 mM glutamine, 100 U/mL penicillin and 0.1 mg/mL streptomycin, and 1% non-essential amino acids. bEnd.3 cells used in all experiments were seeded at a passage of 21–30.

### 4.8. Cellular Uptake and Co-Localizations

For co-localization experiments, 1 × 10^4^ bEnd.3 cells were seeded on round 12 mm-diameter glass coverslips placed inside the wells of a 24-well plate. Cells were incubated for 24 h with 0.1 mg/mL NPs at 37 °C and 5% CO_2_. After incubation, samples were washed two times with PBS to remove non-internalized NPs and were fixed with 4% paraformaldehyde for 20 min. For lysosome staining, the fixed cells were permeabilized with 0.05% saponin-PBS for 10 min and blocked with FBS-PBS 10% for 20 min at room temperature. Lysosomes were localized with rabbit Abcam® anti-LAMP-2 polyclonal primary antibody (Abcam, Cambridge, UK, 1 mg/mL) diluted 1:150 in FBS-PBS 10% after 1 h at room temperature in a humidified chamber. This was completed with Alexa Fluor® 488 anti-rabbit secondary antibody (Abcam, 1 mg/mL) diluted 1:200 in FBS-PBS 10% and incubated for 30 min. The cell nuclei were stained with DAPI (Sigma-Aldrich S.r.l.). Lastly, coverslips were mounted on glass slides with PBS/glycerol (1:1) solution and immunofluorescence analyses were performed by using a confocal laser scanning microscope (Leica TCS SP5 MP, Weitzlar, Germany).

### 4.9. Permeability

bEnd.3 cells were seeded at a density of 3 × 10^4^ cells/cm^2^ on Transwell permeable inserts (6.5 mm diameter, 3 µm pore size, Corning Incorporated, Corning, NY, USA), *n* = 3 inserts were seeded for each sample and the experiments were performed in triplicate. Trans Endothelial Electrical Resistance (TEER) was measured by using Millicell1-ERS voltohmmeter (Millipore, Billerica, MA, USA). Permeability experiments were performed 7 days from the seeding, which allowed sufficient time for the cells to form the tight junctions. On the day of the experiment, the Transwell insert filter was washed with PBS and then the media of the donor chamber was filled with 150 µL cell culture medium without phenol red containing 0.1 mg/mL of NPs while the acceptor chamber is filled with 400 µL of cell culture medium. The samples of 400 µL were drawn every 30 min for 2 h from the acceptor chamber and were then replaced with the same amount of fresh medium. The experiments were performed in triplicate. The fluorescence tracer concentration in the samples was determined by a Victor 2 spectrofluorometer and the excitation and emission wavelengths are set to 557 nm and 578 nm, respectively, for NPs. The permeability *P* of the monolayer was calculated according to the following equation.

(4) $$P=\frac{\frac{\Delta C_{A}}{\Delta t}\times V_{A}}{C_{D}\times S},$$

where $\frac{\Delta C_{A}}{\Delta t}$ was the increase in the fluorescence concentration in the acceptor chamber during the time interval, *C_D_* was the fluorescence concentration in the donor chamber (assumed to be constant during the experiment), *V_A_* was the volume of the acceptor chamber, and *S* was the surface area of the filter.

### 4.10. Glycotech

**Figure 5:**
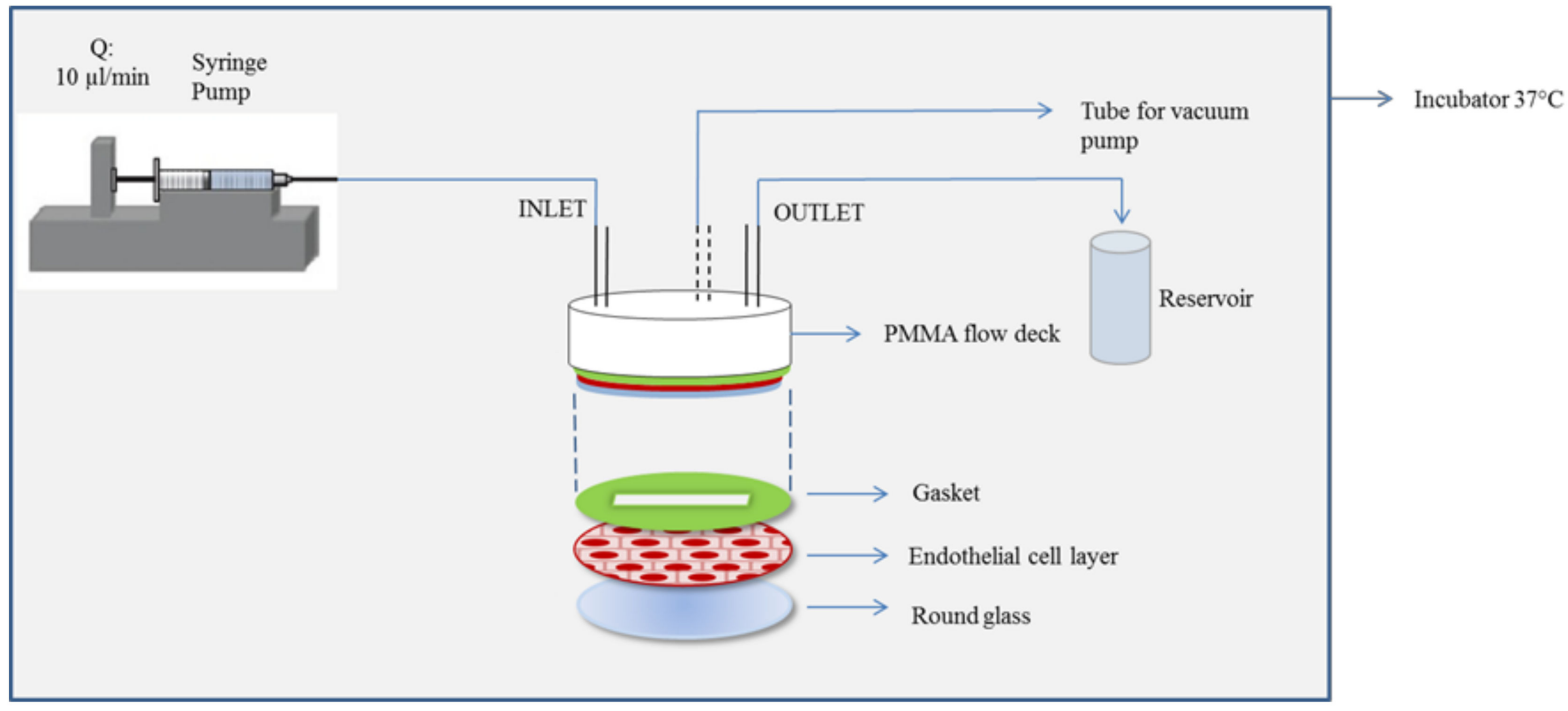
Schematic representation of the Glycotech flow chamber for studying NP interaction with the BBB endothelium.

To investigate the ability of NPs to recognize the in vitro BBB under dynamic condition, 4 × 10^5^ bEnd.3 cells were seeded on round glasses (60 mm diameter) placed in Petri dishes (100 mm diameter). Exactly 3 days from the seeding, the adhesion assays were run by using the Glycotech system. The system consisted of a poly(methyl methacrylate (PMMA) flow deck with inlet and outlet bores, a silicon gasket, and a glass coverslip in Figure 5).

The silicon gasket separated the acrylic flow deck and the glass and defined the flow area. The gasket used in the present experiments had a thickness *h* of 0.01 in and a width *w* of 1 cm. The inlet bore was connected to a syringe-pump through a silastic tube and the outlet bore to a reservoir. The volumetric flow rate *Q* (10 µL/min) was equal for all the experiments. Based on these data, the mean velocity was calculated using Equation (1) within the chamber, the shear rate in Equation (2), and the shear stress at the wall in Equation (3), which had a medium viscosity of 1.2 × 10^−2^ dyn s/cm^2^. The Reynolds number was given by the equation below:

2*whρU_mean_*/[(*w* + *h*)*µ*], (5)

and calculated considering the hydraulic diameter of the chamber 2*wh*/[(*w + h*)] ≅ 500 µm. NP suspensions at the final concentration of 0.2 mg/mL in the cell culture medium supplemented with 40 mM HEPES buffer was flushed by using a 5 mL syringe allocated on a syringe pump. The flow was driven through the parallel plate flow chamber for 1 h at 37 °C in a plexiglass incubator. After the experiment, cells were fixed after two washes with PBS to remove non-adherent NPs. The percentage of the mean gray value was analyzed by using the imageJ 1.44 p software package (Nihuo Software, www.nihuo.com). Data were reported as the percentage of the mean gray value normalized for the mean gray value of 100% fluorescence intensity of NPs.

## 5. Conclusions

In this work, biocompatible nanoparticles functionalized with gH and CRT peptides for targeted drug delivery across the BBB were successfully synthesized. The results clearly indicated that the percentage of functionalization gH/CRT_33/66 was able to target and cross the in vitro cerebral endothelium, significantly. The new nanocarrier gH/CRT_33/66, which is made up of FDA-approved materials, could be considered as a promising strategy for selective and effective administration of therapeutic compounds to the brain. Further work with drug-loaded NPs is underway.

## Figures and Tables

**Table 1:** Hydrodynamic diameter (H_D_) and Z-potential values of the different NP formulations. Data are reported as the mean of three different measurements ± standard deviation (SD).

NP	H_D_ (nm) ± SD	PDI	Z-Potential (mV) ± SD
Blank	88.24 ± 1.62	0.15	−27.40 ± 0.66
CRT	92.06 ± 1.53	0.15	−26.10 ± 1.17
gH	84.53 ± 0.60	0.14	−13.83 ± 0.11
gH/CRT_66/33	80 ± 1	0.16	−22.4 ± 0.5
gH/CRT_50/50	78 ± 2	0.14	−23.8 ± 0.9
gH/CRT_33/66	76 ± 1	0.13	−20.0 ± 0.5

**Table 2:** Amount of co-polymers used for the different NP formulations.

NP	PELGA (mg)	PLGA-Rho (mg)	PLGA-gH (mg)	PLGA-CRT (mg)	PLGA (mg)	mg tot
Blank	1	0.4	-	-	0.8	2.2
gH	1	0.4	0.4	-	0.4	2.2
CRT	1	0.4	-	0.4	0.4	2.2
gH/CRT_66/33	1	0.4	0.4	0.2	0.2	2.2
gH/CRT_50/50	1	0.4	0.4	0.4	-	2.2
gH/CRT_33/66	1	0.4	0.2	0.4	0.2	2.2

## Supplementary Materials

Supplementary materials are available online.

## Abbreviations

Poly(lactic-co-glycolic acid)	PLGA
Polyethylene glycol	PEG
Blood-brain barrier	BBB
Central nervous system	CNS
Receptor-mediated transcytosis	RMT
Transferrin	Tf
Transferrin receptor	TfR
Nanoparticle	NP
Trans Endothelial Electrical resistance	TEER
Ac-CRTIGPSVC-βAK-CONH_2_	CRT
Ac-HGLASTLTRWAHYNALIRAFGGG-COOH	gH
