# Supplementary figures and images for: Design, Synthesis and Characterization of Novel Co-Polymers Decorated with Peptides for the Selective Nanoparticle Transport across the Cerebral Endothelium
Source: Molecules. 2018 Jul 6;23(7):1655. doi: 10.3390/molecules23071655 (PMC6100052; doi:10.3390/molecules23071655)

## Supplementary materials

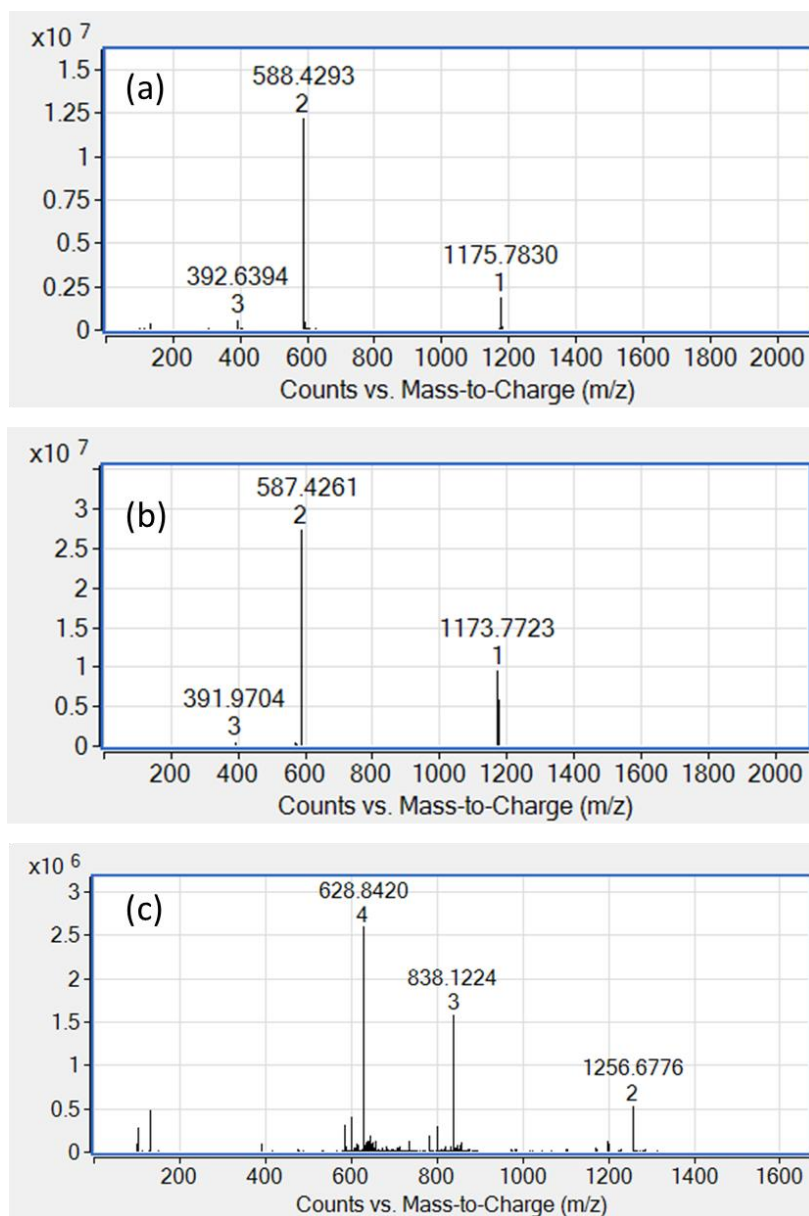

Figure S1: ESI-LC-MS characterization of CRT (a), cyclic CRT (b), gH (c) peptide.

Supplement: Supplementary file 1 [file molecules-23-01655-s001.pdf]
